# Partial Oxidation of CH_4_ in Plasma: The Effects of Oxidant and Catalyst Addition

**DOI:** 10.3390/molecules30091958

**Published:** 2025-04-28

**Authors:** Oleg V. Golubev, Anton L. Maximov

**Affiliations:** A.V. Topchiev Institute of Petrochemical Synthesis, Russian Academy of Sciences (TIPS RAS), Moscow 119991, Russia

**Keywords:** methane partial oxidation, plasma catalysis, methanol from methane, dielectric barrier discharge

## Abstract

The partial oxidation of methane in cold atmospheric plasma represents an innovative and promising approach to energy conversion and sustainable chemical processes for obtaining various chemicals and fuels. In present work, dielectric barrier discharge plasma is applied to the partial oxidation of CH_4_ combined with Cu-containing catalysts. The catalysts with different porous and acidic properties were obtained, characterized by physico-chemical methods and used in plasma-catalytic reaction. The influence of the oxidizing agent (oxygen vs. air) on the products’ selectivity and yield was studied. It was found that using air as an oxidizer was beneficial in terms of CH_4_ conversion and gaseous products yield, as N_2_ aided in denser microdischarge formation. Using pure O_2_ for methane partial oxidation resulted in enhanced oxygenate (mainly CH_3_OH) generation. Furthermore, the Cu-containing catalysts enhanced methanol yield compared to the plasma-only process, as well as the energy efficiency of the process.

## 1. Introduction

Methane, a dominant component of natural gas, is one of the most abundant and economically significant hydrocarbons. In addition to being a crucial source of energy for heating, electricity generation, and transportation, natural gas is increasingly recognized for its potential as a raw material for the production of chemicals and fuels [1,2]. Its direct conversion into value-added products, like syngas, hydrogen, hydrocarbons, and oxygenates, has significant implications for both industrial and environmental applications. However, traditional methods for methane reforming, such as steam methane reforming, often require high temperatures and pressures, leading to high energy consumption and unwanted by-products, like carbon dioxide [3,4,5].

One of the desirable chemicals, which involves natural gas a source, is methanol [6,7]. It is used in a variety of industries, from plastics and pharmaceuticals to fuel additives. The modern industrial method of methanol production from methane is a multistage process involving synthesis gas as an intermediate product [8]. The process is carried out under harsh conditions (temperature at the stage of syngas production from CH_4_ is up to 900 °C, pressure at the stage of methanol production from syngas is up to 100 atm), which determines the high cost of equipment and operating costs [9]. This indicates the need to develop alternative methods for direct production of methanol from methane under mild conditions [10]. Methanol can be produced through the partial oxidation of methane, and this process can be carried out in different ways. Among them, thermochemical [11,12], photochemical [13], electrochemical [14], and plasma-assisted [15,16,17] methods are used. The thermochemical method requires high energy input for heating the gas mixture, involves unstable and expensive oxidants (H_2_O_2_), and is characterized by low methanol yield, based on published work [18]. The electrochemical method has the following disadvantages: low solubility of gases in the electrolyte (use of highly dilute solutions) and instability of the process due to the deactivation of electrodes. The conversion of methane to methanol through the photochemical method also has limitations due to very low gas concentrations in the working solution and the difficulty of separating the products from the solution. Another alternative way to carry out partial oxidation of methane is the plasma-catalytic approach. The CH_4_ molecule is stable due to its high C-H bond energy (439 kJ/mol), which in the conventional process requires heating to a high temperature to activate it. In contrast, cold plasma technology offers a more energy-efficient and environmentally friendly alternative by enabling chemical reactions to occur under milder conditions—at or near room temperature—without the need for thermal heating, which helps in minimizing the thermal decomposition of methane [19].

The combination of energetic species in the plasma facilitates the activation of methane molecules, allowing for the partial oxidation process, where methane reacts with oxygen to produce syngas and other valuable products, like formaldehyde, acetylene, or C₂ hydrocarbons [20,21]. These reactions are typically carried out in dielectric barrier discharge (DBD) reactors, a widely studied configuration for methane plasma activation due to its ability to generate stable and controlled plasma fields at atmospheric pressure and ambient temperature [19].

To increase the efficiency of the process in plasma, a catalyst is introduced into the reactor, which allows it, in some cases, to increase the conversion of the reacting gas and selectivity for the target product. There is a large number of plasma-catalytic reactions, which are described in the literature, including, for example, CO_2_ decomposition in the presence of CeO_2_ [22,23,24], CO_2_ hydrogenation with methanol production [25,26], dry methane reforming with oxygenate formation [27,28], and many others. It should be noted that the interactions between the catalyst, plasma (also known as plasma–catalyst synergism), and the reaction species are quite complex and require further understanding. When the catalyst particles are exposed to plasma species, their physico-chemical properties may change, such as the oxidation state, surface characteristics, and coking resistance [29]. The presence of the catalyst in the discharge zone also affects the physical properties of the plasma [30,31,32]. Thus, in the presence of heterogeneous particles in the plasma, microdischarges of various types occur between their surfaces, which influence the dissociation rate of molecules and radicals in the gas phase [33]. The use of various types of heterogeneous catalysts in a barrier discharge reactor for the production of methanol from methane is reported. Among them are catalysts based on platinum group metals (Pd, Pt [34,35]), zeolite-containing catalysts (Na-ZSM-5, Cu-ZSM-5 [36], as well as those deposited on aluminum oxide support (Cu/ZnO/Al_2_O_3_ [37], Fe_2_O_3_-CuO-Al_2_O_3_ [38], Cu-Al_2_O_3_, Ni-Al_2_O_3_, Fe-Al_2_O_3_ [39]). It is noted that the most promising is the use of copper compounds as an active component due to high activity and selectivity for methanol.

While plasma-catalytic oxidation of methane holds great potential, several challenges need to be solved. There remains a wide space for research in this area related to the influence of the support as well as various promoters on the efficiency of the catalyst. In addition to the influence of the catalyst, oxidant composition can affect the partial oxidation process of methane. Thus, when air is used as an oxidizer, an increase in methane conversion is observed with a simultaneous decrease in methanol selectivity [40] in a barrier discharge reactor without the use of a catalyst.

This works aims to investigate the influence of porous characteristics as well as oxidation agent influence on CH_4_ conversion and product distribution. For this purpose, Cu-based catalysts with various porosities (micro, meso, and macro) were paired with a DBD reactor for the low-temperature partial oxidation of methane. Physico-chemical properties of the catalysts were investigated, gaseous and liquid products’ yields were calculated, and the characteristics of the discharge were studied. As for oxidant variation, pure O_2_ and compressed ambient air were taken for the partial oxidation of CH_4_.

## 2. Results

### 2.1. Plasma-Catalytic Methane Partial Oxidation

**Figure 1:**
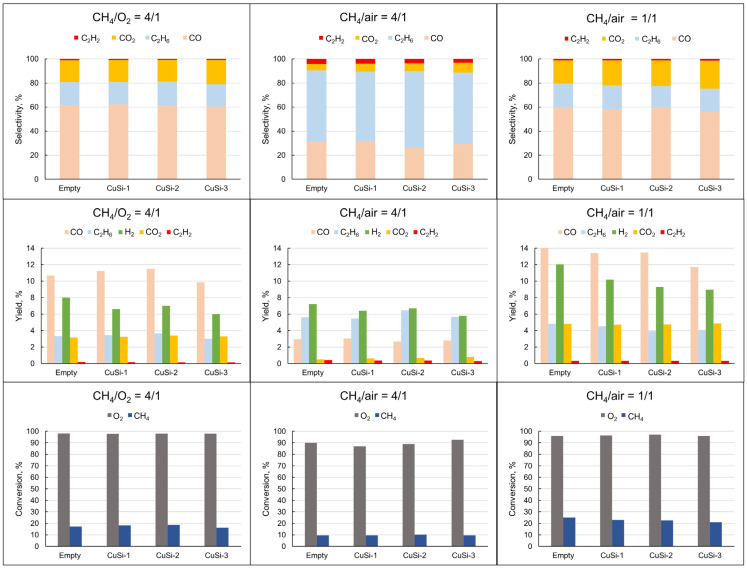
The results of plasma and plasma-catalytic methane partial oxidation.

The evaluation of methane partial oxidation was carried out using three types of Cu-containing catalysts, which were impregnated on microporous (CuSi-1), mesoporous (CuSi-2), and macroporous (CuSi-3) supports. At the same time, different gas reaction mixtures were used, which included O_2_ and air with various CH_4_/air ratios. The results of the plasma-catalytic experiments are shown in Figure 1.

The main gaseous reaction products were H_2_, CO, C_2_H_6_, and CO_2_, with a little amount of C_2_H_2_. It is seen that the highest CH_4_ conversion as well as the highest product yields were achieved using (CH_4_/air = 1/1) gas mixture as a feed. The distribution of the products (selectivity) in the case of (CH_4_/O_2_ = 4/1) and (CH_4_/air = 1/1) mixtures was quite similar, in contrast to the (CH_4_/air = 4/1) mixture. In the case of the latter gas mixture, the selectivity of C_2_H_6_ was the highest among the C-containing gases, and the yield of H_2_ was the highest. It is also remarkable that the selectivity and the yield of acetylene were higher than those in the case of (CH_4_/O_2_ = 4/1) and (CH_4_/air = 1/1) mixtures. With the addition of the catalyst, H_2_ yield (as well as CO yield in the case of the (CH_4_/air = 1/1) mixture) was slightly lowered compared to that in the absence of any catalyst (empty reactor).

**Figure 2:**
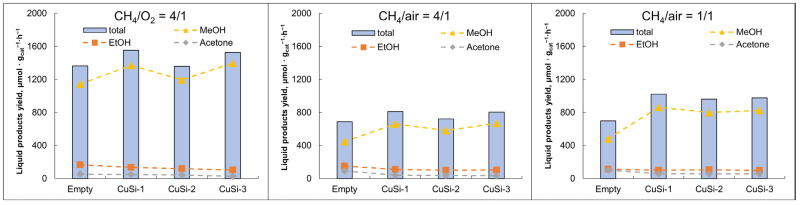
Distribution of the liquid products and the total liquid quantity in the sample after 90 min of the reaction. MeOH = methanol, EtOH = ethanol.

The liquid products’ mixture was obtained through the barbotage of the effluent through the ice-chilled beaker filled with ethyl acetate as a solvent. The main products of the liquid phase were methanol, ethanol, and acetone. From Figure 2, it is seen that the highest yield of liquid products was achieved in the presence of the (CH_4_/O_2_ = 4/1) mixture using the CuSi-1 catalyst. It is also pointed out that the ethanol and acetone quantities remained almost unchanged when the catalyst was present, but the methanol yield was enhanced in the presence of the catalyst. It is also seen that when using pure O_2_ as an oxidizing agent, the maximum methanol yield was obtained.

**Figure 3:**
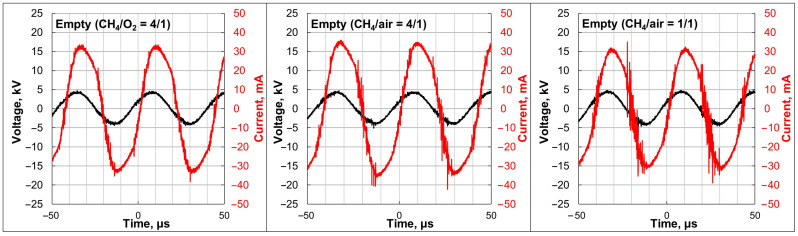
Voltage–current oscillograms utilizing different gas mixtures as a feedstock.

The data in Figure 3 illustrate how different gas compositions influenced the electrical characteristics of the DBD. According to the measured electric signals, the voltage was constant at ~5 kV, and the amperage was ~30 mA. In the case of pure O_2_ as an oxidizer (CH_4_/O_2_ = 4/1), a few microdischarges were seen on the current oscillogram. When air was used as an oxidizer, the density of the microdischarges was enhanced, and it was highest in the case of the (CH_4_/air = 1/1) mixture.

**Figure 4:**
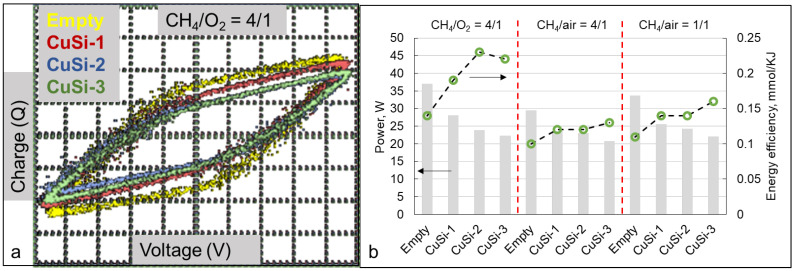
Lissajous figures (**a**) and power and energy efficiency diagrams (**b**).

Lissajous figures were also obtained using an oscilloscope and used to determine the input power of the DBD. From Figure 4a, it is seen that the Lissajous figures represented a parallelogram, which is typical for DBD. The biggest area enclosed by the figure was in the case of the empty reactor (without the catalyst). When the samples of CuSi-1–CuSi-3 were present in the reactor, the area of the Lissajous figure was decreased, and the corresponding input power value was also lower than in the case of the empty reactor (Figure 4b). The energy efficiency of the reactor was enhanced in the presence of the catalysts, and it was the highest (0.22 mmol/kJ) in the case of the (CH_4_/O_2_ = 4/1) gas mixture with CuSi-2 as a catalyst.

### 2.2. Catalysts’ Characterization

The catalysts obtained were characterized using a variety of physico-chemical methods. Through X-ray fluorescent spectroscopy analysis (XRF), elements were determined and calculated as oxides in the samples (Table 1).

**Figure 5:**
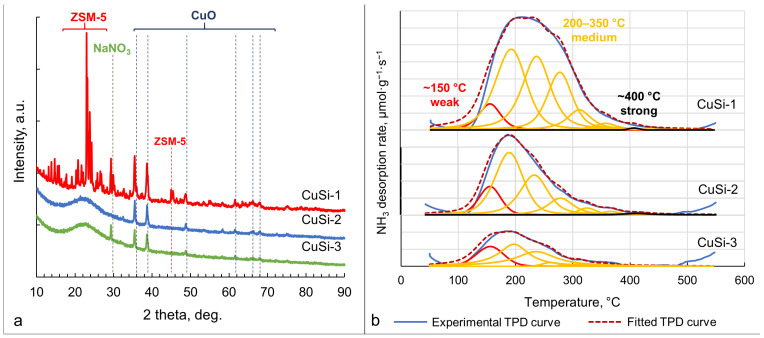
(**a**) Diffractograms of the obtained samples; (**b**) NH_3_-thermoprogrammed desorption profiles.

X-ray diffraction patterns (Figure 5a) contained the signals of ZSM-5 zeolite (PDF card #44-0003, for CuSi-1 sample), CuO (PDF card #48-1548, for all samples), and NaNO_3_ (PDF card #36-1474, for CuSi-3 sample). The latter compound is present in the CuSi-3 sample due to incomplete washing of Na^+^ during the synthesis of meso- and macroporous silica supports.

The acidity of the samples was evaluated through NH_3_-TPD analysis (Figure 5b). The pattern can be distinguished by weak acid sites (~150 °C), medium acid sites (~200–350 °C), and strong acid sites (~400 °C). According to the literature, the low-temperature adsorption is attributed to physically adsorbed ammonia on weak acid sites [41]. Therefore, the true acid sites, which are medium and strong acid sites, correspond to the moderate- and high-temperature peaks (Table 2). CuSi-1 catalysts possessed the highest concentration of acid sites due to the zeolite support (ZSM-5). The concentration of acid sites in the CuSi-3 sample was the lowest among other catalysts.

**Figure 6:**
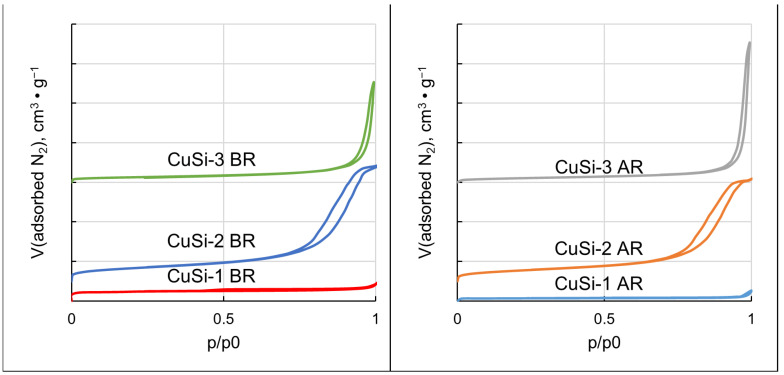
Low-temperature N_2_ adsorption–desorption isotherms of catalysts before the reaction (BR) and after the reaction (AR).

Adsorption–desorption isotherms obtained from low-temperature N_2_ adsorption analysis are shown in Figure 6. The isotherm of the CuSi-1 sample, according to the IUPAC classification [42], corresponds to type I, which indicates the microporosity of the zeolite ZSM-5. The isotherms of the CuSi-2 and CuSi-3 samples correspond to type IV with a hysteresis loop, which is characteristic of mesoporous and macroporous materials. The hysteresis loop shape H3 is observed for the CuSi-3 sample, which is characteristic of macroporous materials, while the hysteresis loop of the CuSi-2 sample belongs to type H1, which is typical for open mesopores [43]. The shape of the isotherms in the case of the samples after the reaction remained unchanged, which indicated that the structure of the supports was stable under the DBD conditions.

Surface characteristics of the samples before and after reactions are summarized in Table 3. It is seen that the surface area of the CuSi-1 sample was reduced drastically after the reaction. The same phenomenon to a lesser extent is observed for the CuSi-2 and CuSi-3 samples.

## 3. Discussion

### 3.1. Plasma-Catalytic Methane Partial Oxidation

The discharge characteristics differed when air was used as an oxidizer instead of pure O_2_. This was related to the presence of N_2_ contained in air and its concentration in the mixture. When the N_2_ gas concentration was ~15 vol% (CH_4_/air = 4/1 mixture), denser microdischarges (vertical peaks on the sinusoidal wave) were observed in the oscillogram of the current (Figure 3). When the N_2_ concentration was ~45 vol% (CH_4_/air = 1/1 mixture), many more peaks were present on the oscillogram. As explained in [44,45], changing the gas composition sufficiently affects the physical characteristics of the plasma, including gas temperature, reduced electric field strength, and average electron energy. Thus, the N_2_ addition (contained in air) resulted in more active plasma species, which gave more CH_4_ dissociation impact. Consequently, CH_4_ conversion was the highest in the case of the (CH_4_/air = 1/1) mixture, which is seen in Figure 1. The higher CH_4_ conversion also led to higher gaseous product yields in comparison to the (CH_4_/O_2_ = 4/1) mixture. Remarkably, the selectivity of the gaseous products was quite similar in the case of (CH_4_/O_2_ = 4/1) and (CH_4_/air = 1/1) gas compositions, which was explained by the similar ratio of CH_4_ and oxygen in both cases. Different product distributions in the case of the (CH_4_/air = 4/1) mixture resulted from the predominant CH_4_ quantity, which gave rise to the yields of C_2_H_6_ and C_2_H_2_ and lowered the CO and CO_2_ yields. In DBD plasma, electron collisions with CH_4_ molecules give CH_3_· radicals (CH_4_ +e^−^ → CH_3_· + H· + e^−^), CH_2_· radicals (CH_4_ +e^−^ → CH_2_· + H_2_ + e^−^), and CH· radicals (CH_4_ +e^−^ → CH· + H· + H_2_ + e^−^) [21]. In the case of the CH_4_-rich mixture, more electron-activated radicals CH_3_· recombined with the formation of ethane (CH_3_· + CH_3_· → C_2_H_6_) and acetylene (CH· + CH· → C_2_H_2_), which is evident from the selectivity plot. In cases with more O_2_ concentration in the mixture (CH_4_/O_2_ = 4/1 and CH_4_/air = 1/1 mixtures), reactive O· species, which are produced from the electronic collision with O_2_, react with the CH_3_· and H· radicals, giving such reactive species as OH, HO_2_·, CH_3_O·, CH_3_O·, and CH_3_OO·. These radicals (CH_3_OO·) further react with CH_3_· to form CH_3_O·, which consequently results in methanol formation (CH_3_O· + CH_4_ → CH_3_OH + CH_3_·) [20].

In Figure 2, the methanol yield tends to increase in the presence of the catalysts, which is due to Cu active sites of the catalysts. As known from the literature, Cu has a strong oxidizing capability, and copper oxides are known to facilitate CH_4_ combustion with methanol formation [20]. As mentioned above, various reactive species are formed in plasma, including CH_x_· radicals (x = 1, 2, 3), O·, and OH·. These radicals are adsorbed onto the surface of the catalyst and bind the active sites (Cu^2+^), with the subsequent formation of Si-Cu-OCH_3_ and Si-Cu-OHCH_3_ species [36]. After stepwise hydrogenation with CH_3_OH formation, methanol is desorbed from the catalyst surface, and the catalytic cycle is repeated [39]. Due to the presence of more acid sites on its surface, not only methanol production increases but also selectivity towards C2 oxygenates is enhanced, which is confirmed by the liquid product results. In the presence of CuSi-1 (as a catalyst with more acid sites among other samples), a slightly higher amount of ethanol and acetone was produced compared to CuSi-2 and CuSi-3 samples (Figure 2).

Liquid product yield was reverse correlated with gaseous product yield. Thus, in the presence of the CuSi-2 sample, the highest gaseous product yields and the lowest liquid product yields were achieved. We relate this observation to the largest surface area of the CuSi-2 sample (234 m^2^/g) among the other catalysts, which may lead to enhanced adsorption of the reactive species and further over-oxidation of the CH_3_OH to CO and CO_2_, which is obviously an undesirable reaction path in the methane-to-methanol process. It is stated in [46] that the strong adsorption of the CH_3_OH molecule is the key factor inhibiting CH_3_OH selectivity in plasma-catalytic methane partial oxidation to methanol.

It should be noted that in the presence of the catalysts, the measured input power was lower in comparison with that in an empty reactor (plasma-only mode, Figure 4a). When the catalyst was placed into the reactor, the discharge characteristics were affected, and the Lissajous figure area tended to decrease. Due to this fact, despite the comparable CH_4_ conversion in the presence of the catalysts, energy efficiency was much higher, and thus a smaller amount of energy was demanded to achieve the same CH_4_ conversion.

The obtained results were compared with the previously published ones on plasma-catalytic partial methane oxidation, and they are summarized in Table 4. It should be noted that the direct comparison of the results is complicated due to the non-unified reactor configuration (various electrode materials, discharge gaps, discharge lengths, and catalyst positions in the discharge zone) and working parameters (input power, discharge frequency, total gas flow, CH_4_/O_2_ ratio, catalyst quantity, etc.). However, the general observations can be discussed. It was confirmed from the literature that the Cu active sites promote the formation of oxygenates, mainly C1 oxygenates (methanol, formaldehyde, and formic acid) with the additional formation of C2+ oxygenates (e.g., ethanol, acetic acid, and acetone), which is consistent with the present study. It is seen from Table 4 that power consumption falls within a wide range, generally indicating that applying higher input power enhances CH_4_ conversion. However, energy efficiency will be lower in such cases. The conversion of CH_4_ and methanol selectivity trade-off are hard to achieve. Obtaining reasonable CH_4_ conversion will most likely result in low CH_3_OH selectivity and vice versa [47]. For example, the highest methanol yield in the present work was 1370 μmol·g_cat_^−1^·h^−1^, which was almost an order of magnitude lower than that in [47]. However, the conversion of CH_4_ was comparably low in the above-mentioned paper. Thus, further research on catalyst composition is needed to implement efficient plasma-catalytic direct methane oxidation to methanol. 

### 3.2. Catalysts’ Characterization

As can be seen from the XRF, CuO content was close to the calculated amount. It is noteworthy that a significant amount of Na was determined in all samples. The presence of Na in the CuSi-1 sample is related to Na^+^ ions in the structure of the parent zeolite, and the presence of Na^+^ in the CuSi-2 and CuSi-3 samples is related to the NaNO_3_, which was produced during the synthesis of the supports. Thus, a more rigorous washing procedure is needed to eliminate the Na^+^ ions. It is also confirmed through XRD analysis that the NaNO_3_ phase was present in the CuSi-3 sample (Figure 5a).

The comparison of the adsorption isotherms before and after the reaction (Figure 6) showed that the structure of the support materials did not change, but the surface area of the samples was reduced, with the most drastic reduction in the case of the CuSi-1 sample. We can relate this phenomenon to smaller CuSi-1 pores, which were blocked by the resin-like compounds, which was seen on the quartz tube surface in the outlet of the discharge zone (Figure S1). The tube coating was analyzed through gas chromatography, and different oxygenates were identified, mainly, butanol-1 and ethylene glycol. We assume that the observed resin coating was the product of partial polymerization or oligomerization of the named alcohols. However, it was not possible to identify these compounds by means of gas chromatography. The assumption of the higher structures’ blockage of the pores was confirmed by thermogravimetric analysis of the samples after the reaction (Figure S2). It was found that ~6–8% of the mass loss was in the temperature range of 280–305 °C, which indicated the presence of the components with higher molecular mass. The high acidity of the CuSi-1 sample may also contribute to the formation of such components. However, we may consider this effect undesirable, as higher molecules deactivate the catalyst and it needs to be further regenerated.

Overall, it can be concluded that the catalysts’ composition played a minor role in the studied conditions (except for methanol yield enhancement and enhancement of the energy efficiency of the process). The effect of the oxidizer mixture and the CH_4_/oxidizer ratio played more pivotal roles in tuning gaseous products’ selectivity and CH_4_ conversion than the structure and the properties of the catalysts. Nevertheless, further research of the catalyst is needed to enhance the yield of oxygenates and the conversion of CH_4_. Among different approaches, the bulk Cu catalysts (without need of the support) can be utilized, and the promotors can be added to the catalysts’ composition. Thus, future research regarding plasma-catalytic partial oxidation of methane will be focused on further catalyst modification.

## 4. Materials and Methods

### 4.1. Catalysts’ Synthesis

For the supports of the catalysts, different porous materials were taken, which are summarized in Table 5. The mesoporous and macroporous supports were synthesized using the same synthesis procedure (except for the reagents’ addition order) from inorganic inexpensive reagents to ensure its low cost and scalability in future. Zeolite ZSM-5 (JSC “NZHK”, Novosibirsk, Russia) was chosen as a microporous support due to its commercial availability and well-known microporous structure. The detailed description of mesoporous and macroporous supports synthesis is given in the Supplementary Materials. The obtained supports were impregnated with the solution of Cu(NO_3_)_2_·3H_2_O (JSC “Lenreaktiv”, Saint Petersburg, Russia) through the wetness impregnation technique. The calculated amount of deposited CuO was 10 wt%.

### 4.2. Physico-Chemical Methods

X-ray diffraction (XRD) patterns were recorded using the TD-3700 X-ray diffractometer (Tongda Science & Technology Co., Ltd., Dandong, China). The device was equipped with a copper anode X-ray tube, a Mythen2R 1K (DECTRIS Ltd., Baden-Dättwil, Switzerland) linear multichannel semiconductor detector, and a Goebel mirror for parallel beam formation. Data were collected over a 2θ range of 10–90°.

The elemental composition of the catalysts was determined using the ARL PERFORM′X Sequential X-Ray Fluorescence Spectrometer (Thermo Fisher Scientific, Ecublens, Switzerland). with an X-ray tube power of 2500 W.

The specific surface area (S_BET_), pore volume V(pores), and pore diameter d(pores) of the catalysts were measured using the BELSORP MINI X analyzer (Microtrac MRB, Osaka, Japan). Prior to analysis, samples underwent thermal degassing at 300 °C for 8 h under a pressure of 10 Pa. The Brunauer–Emmett–Teller (BET) method was employed to calculate the specific surface area within a relative pressure range (p/p_0_) of 0.05–0.2.

The acidity of the samples was measured through NH_3_ temperature-programmed desorption (NH_3_-TPD) using a USGA-101 (LLC “Unisit”, Moscow, Russia) device. The sample (m = 0.15–0.2 g) was treated in He flow (T = 512 °C for 40 min) and subsequently saturated with NH_3_ (5 vol% NH_3_–95 vol% He) at 60 °C for 24 min. The analysis was conducted in He flow at 100–800 °C (heating rate 7 °C/min). Desorbed NH_3_ registration was carried out using a thermal conductivity detector. The TPD profiles were deconvoluted using PeakFit (v4.12) software.

Thermogravimetric analysis with differential scanning calorimetry (TGA) was conducted using the TGA/DSC 3+ analyzer (Mettler Toledo, Columbus, OH, USA) in the temperature range of 30–900 °C in the air atmosphere.

### 4.3. Plasma-Catalytic Experiments

**Figure 7:**
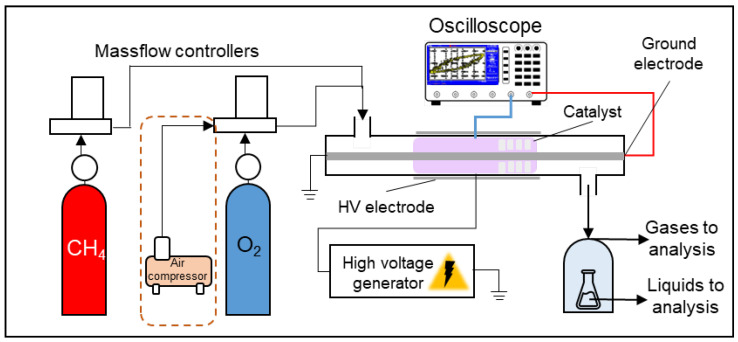
A scheme of the laboratory plasma-catalytic unit.

The process of partial methane oxidation was carried out using a laboratory plasma-catalytic unit with a DBD reactor (Figure 7). The reactor comprised a quartz tube (16 mm outer diameter, 2 mm wall thickness, 160 mm length) serving as the dielectric barrier. A steel rod (8 mm diameter) with threading functioned as the ground electrode inside of the reactor, while a steel mesh (0.5 mm mesh size, 80 mm length) wrapped around the outside wall acted as the high-voltage electrode. The discharge gap was 4 mm. A catalyst sample (m = 1 g) was placed within the reactor and fixed with quartz wool.

A mixture of gases consisted either of CH_4_ (99.995%)/O_2_ (99.999%) or CH_4_/air (compressed with the air compressor) and was introduced into the reactor using RRG-20 (LLC “Eltochpribor”, Zelenograd, Russia) mass flow controllers. The flowrate was 50 mL/min. The high-voltage power source supplied a sine wave signal at a frequency of 23 kHz. Discharge voltage, current, and Lissajous figures were monitored using a TDS 2012B oscilloscope (Tektronix, Beaverton, OR, USA). Based on the Lissajous figure, the plasma absorbed power was calculated according to the equation

(1) $$P\;(W)=fC_{n}A$$

where *C_n_* is the value of the capacitor included in series with the discharge tube, *f* is a frequency of the applied voltage, and *A* is the area of a Lissajous figure.

The energy efficiency of the process (*η*) was calculated as a ratio of converted CH_4_ to the absorbed power using the following equation:

(2) $$\eta \left(mmol\times {kJ}^{-1}\right)=\frac{\nu_{conv}}{P}\times \frac{1000}{60}\;,$$

where $\nu_{conv}$ is the quantity of the CH_4_ converted (mol/min) and *P* is the absorbed power (W).

Gaseous products were analyzed using a PIA gas chromatograph (LLC “NPF MEMS”, Samara, Russia) equipped with a thermal conductivity detector. The chromatograph featured a Hayesep N adsorbent column (2 m length) and a molecular sieves 13Å column (2 m length) for effective separation and analysis of the reaction products. Liquid products were analyzed using Trace GC Ultra chromatograph (Thermo Fisher Scientific, Ecublens, Switzerland) with a flame ionization detector. The mass content of reaction liquid products in the sample was determined through quantitative chromatographic analysis using the internal standard method with Octanol-1 (99.5%, Shanghai Macklin Biochemical Co., Ltd., Shanghai, China) as a standard.

Based on the data obtained during the chromatographic analysis, the conversion (*X*), product selectivity (*S*), and product yield (*Y*) were calculated according to the equations

(3) $$X_{{CH}_{4}}\left(\%\right)=\frac{\nu_{{CH}_{4}\left(in\right)}-{\;\nu }_{{CH}_{4}\left(out\right)}}{\nu_{{CH}_{4}\left(in\right)}}\times 100\%$$

(4) $$X_{O_{2}}\left(\%\right)=\frac{\nu_{O_{2}\left(in\right)}-{\;\nu }_{O_{2}\left(out\right)}}{\nu_{O_{2}(in)}}\times 100\%$$

(5) $$S_{CO}\left(\%\right)=\frac{{\;\nu }_{CO\left(prod\right)}}{\nu_{{CH}_{4}\left(conv\right)}\;}\times 100\%$$

(6) $$S_{{CO}_{2}}\left(\%\right)=\frac{{\;\nu }_{{CO}_{2}\left(prod\right)}}{\nu_{{CH}_{4}\left(conv\right)}\;}\times 100\%$$

(7) $$S_{H_{2}}\left(\%\right)=\frac{{\;\nu }_{H_{2}\left(prod\right)}}{2\times \nu_{{CH}_{4}\left(conv\right)}\;}\times 100\%$$

(8) $$S_{C_{2}H_{6}}\left(\%\right)=\frac{{\;2\times \nu }_{C_{2}H_{6}\left(prod\right)}}{\nu_{{CH}_{4}\left(conv\right)}\;}\times 100\%$$

(9) $$S_{C_{2}H_{2}}\left(\%\right)=\frac{{\;2\times \nu }_{C_{2}H_{2}\left(prod\right)}}{\nu_{{CH}_{4}\left(conv\right)}\;}\times 100\%$$

(10) $$Y\left(\%\right)=\frac{S\times X_{{CH}_{4}}}{100}$$

where $\nu_{{CH}_{4}\left(in\right)}$ is the quantity of CH_4_ injected into the reactor (mol), ${\;\nu }_{{CH}_{4}\left(out\right)}$ is the quantity of the gas in the outlet stream (mol), ${\;\nu }_{\left(prod\right)}$ is the quantity of the gas produced (mol), $\nu_{\left(conv\right)}$, and is the quantity of CH_4_ converted into the products during the reaction.

## 5. Conclusions

In this work, methane partial oxidation was carried out in DBD plasma combined with Cu catalysts, and the influence of the oxidizing agent and the catalyst on process effectivity was evaluated. It was stated that the N_2_, which was contained in air, contributed to the production of more active plasma species, which gave more CH_4_ dissociation impact. It resulted in enhanced CH_4_ conversion and a greater yield of gaseous products. When the CH_4_ to oxygen ratio was the highest (CH_4_/air = 4/1 mixture), less carbon oxides (CO, CO_2_) were produced, and more methane coupling products (C_2_H_6_, C_2_H_2_) were produced. Introducing the Cu catalyst into the discharge zone resulted in enhanced oxygenate yield, mainly methanol; however, the gaseous products’ yield (H_2_) was lowered. Mesoporous silica supported Cu catalyst was less active in methanol formation and more active in gaseous products’ formation compared to other catalysts, which is attributed to the hindered desorption of methanol from the catalyst surface and the subsequent over-oxidation to CO and CO_2_. Moreover, process energy efficiency in the presence of the catalyst was greater than in the empty reactor (plasma-only mode). Future research will focus on catalyst composition modification. The investigation of the key factor determining maximum oxygenates’ yield is pivotal for the methane to methanol plasma-catalytic process.

## Figures and Tables

**Table 1:** Oxide content determined through X-ray fluorescent spectroscopy analysis.

Sample	Oxide Content, wt%			
	SiO_2_	CuO	Na_2_O	Al_2_O_3_
CuSi-1	80	12.8	4	3.2
CuSi-2	87.1	10.1	2.8	-
CuSi-3	84.2	11.3	4.5	-

**Table 2:** Quantity of the acid sites determined through NH_3_–TPD.

Sample	Acid Site Concentration, μmol/g			
	Weak Sites ~150 °C	Medium Sites ~200–350 °C	Strong Sites ~400 °C	Total (Medium + Strong Sites)
CuSi-1	13	153	4	157
CuSi-2	12	83	1	84
CuSi-3	8	43	0	43

**Table 3:** Surface characteristics of the catalysts before (BR) and after (AR) the plasma-catalytic reaction.

Sample	S_BET_, m^2^/g	V_pores_, cm^3^/g	d(pores), nm
CuSi-1 BR	180	0.11	1.4
CuSi-1 AR	50	0.05	0.8
CuSi-2 BR	234	0.87	13
CuSi-2 AR	190	0.79	13
CuSi-3 BR	89	0.61	52
CuSi-3 AR	73	0.92	57

**Table 4:** The comparison between the different Cu-containing catalytic compositions’ performance in plasma-catalytic partial oxidation of methane known from the literature (n/d = no data).

Catalyst Composition	*X*(CH_4_), %	CH_4_/O_2_ Ratio	Oxygenates’ Distribution	Power, W	Remarks	Ref
Cu/γ-Al_2_O_3_	12.5	5/1	HCOOH, CH_3_OH, HCOH, C_2_H_5_OH, CH_3_COOH, CH_3_C(O)CH_3_	1.8	The acid sites on the surface increase the C2 oxygenates’ formation	[39]
CuO/γ-Al_2_O_3_	35	4/1	CH_3_OH	61	Mo promoter reduces CO_2_ selectivity	[48]
Mo-CuO/γ-Al_2_O_3_	31					
CuO-ZnO/Al_2_O_3_	25	4/1	CH_3_OH, HCOH	50	Syngas yield decreased with catalyst addition	[49]
Cu-ZnO/Al_2_O_3_	n/d	5/1	CH_3_OH	60–80	CuO resulted in higher methanol selectivity than that of CuO	[37]
Fe_2_O_3_-CuO/ceramic pellet	25	1/1	CH_3_OH	140	The CuO promoter had no significant effect on methane conversion but enhanced methanol selectivity	[38]
Fe_2_O_3_-CuO/γ-Al_2_O_3_	43	1/1	CH_3_OH	120	Higher methanol yield was observed using the “In plasma catalysis” configuration	[50]
ZSM-5	6	4/1	HCOH, CH_3_OH	20	Formaldehyde was the main product	[51]
Cu/MOR	8	4/1	HCOH, HCOOH CH_3_OH	11–14	Higher loading of Cu decreased methanol selectivity; wetness-impregnated Cu catalysts led to over-oxidation of CH_4_ to CO_2_	[47]
Cu-ZSM-5	6	4/1	HCOH, HCOOH CH_3_OH	15	Dealumination of the zeolite resulted in methanol yield increase	[36]
Cu- ZSM-5 (dealuminated)	6					
Cu/microSiAl	23	4/1	CH_3_OH, C_2_H_5_OH, CH_3_C(O)CH_3_	26	Less methanol yield and more gaseous product yield was observed in the presence of mesoporous silica supported sample	This work
Cu/mesoSi	22.5			24		
Cu/macroSi	21			22		
CuZrAl	9	7.5/1	HCOH, CH_3_OH	1.7	Promoters enhanced the oxygenate yield due to increased dispersion of Cu particles	[52]
CuZnAl						
CuMgAl						

**Table 5:** Different supports used for catalyst synthesis.

Sample	Support	
CuSi-1	Microporous	Purchased Zeolite ZSM-5
CuSi-2	Mesoporous	Synthesized
CuSi-3	Macroporous	Synthesized

## Data Availability

Data are contained within the article.

## Supplementary Materials

The following supporting information can be downloaded at https://www.mdpi.com/article/10.3390/molecules30091958/s1, Figure S1: Resin-like compounds produced in the outlet side of the reactor; Figure S2: TGA data of the catalysts after the reaction; Figure S3: Pore size distribution based on desorption branch of the BJH plot of mesoporous and macroporous supports.

## Abbreviations

The following abbreviations are used in this manuscript:

BET	Brunauer–Emmett–Teller
DBD	Dielectric barrier discharge
TGA	Thermogravimetric analysis
TPD	Temperature-programmed desorption
XRD	X-ray diffraction
XRF	X-ray fluorescent spectroscopy analysis
