# Supplementary material for: Partial Oxidation of CH4 in Plasma: The Effects of Oxidant and Catalyst Addition
Source: Molecules. 2025 Apr 28;30(9):1958. doi: 10.3390/molecules30091958 (PMC12074086; doi:10.3390/molecules30091958)
Supplement: Supplementary file 1 [file molecules-30-01958-s001.zip › molecules-3557453-supplementary.pdf]

molecules

Article

Partial Oxidation of CH_4 in Plasma: The Effects of Oxidant and Catalyst Addition

Oleg V. Golubev * and Anton L. Maximov

A.V. Topchiev Institute of Petrochemical Synthesis, Russian Academy of Sciences (TIPS RAS), Moscow 119991, Russia
* Correspondence: golubev@ips.ac.ru

Supplementary materials

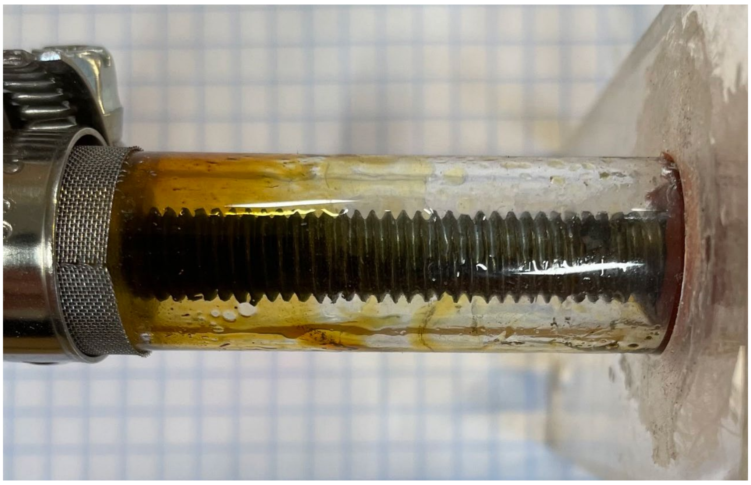

Figure S1. Resin-like compounds produced in the outlet side of the reactor.

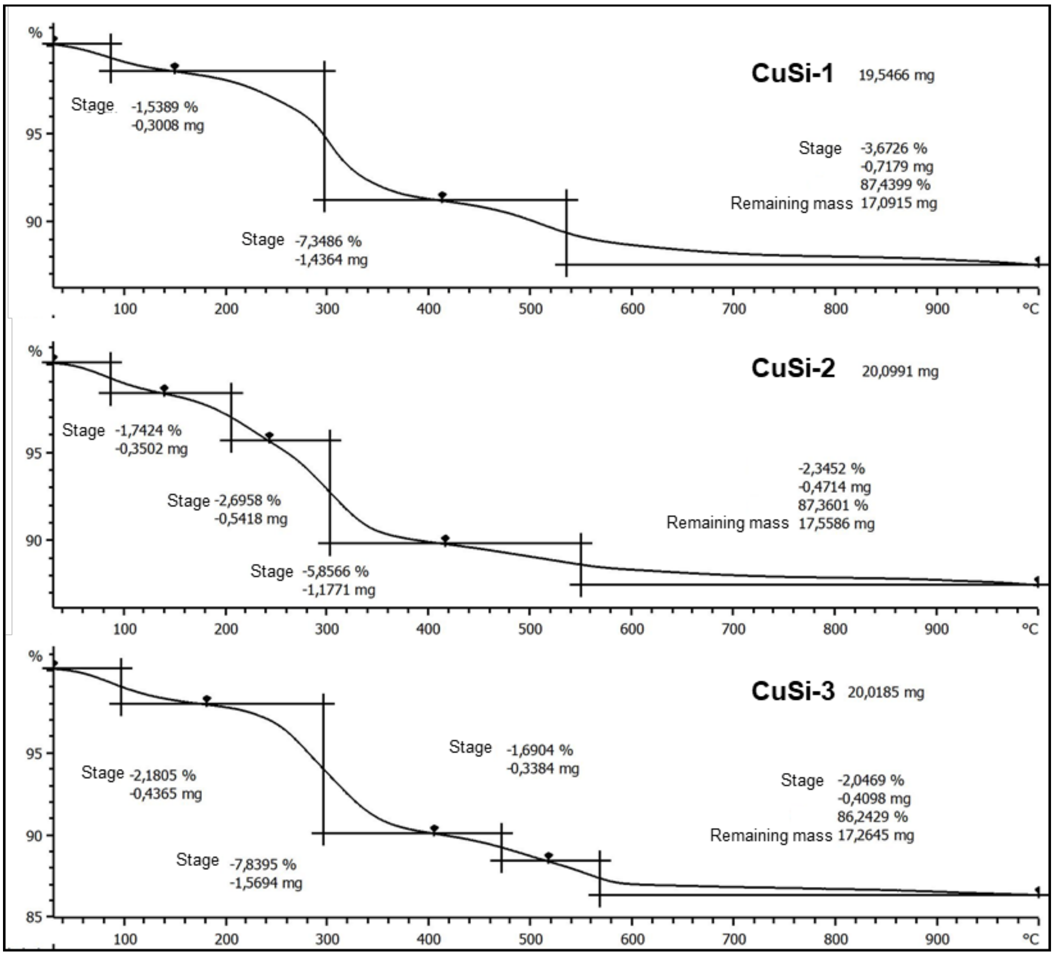

Figure S2. TGA data of the catalysts after the reaction.

Mesoporous and macroporous support synthesis

Mesoporous SiO_2 support was prepared according to the following procedure. 26.4 g of Na_2SiO_3*$9H_2O$ (>98%, LLC Himprom-m, Russia) were dissolved in 83 ml of distilled water. Obtained solution was added dropwise to the 3M HNO_3 solution until pH of the solution was 7. With approaching to the pH = 7, the solution was turning into gel. The mixture was then transferred to the teflon-lined stainless steel autoclave reactor and aged for 20 h at 60°C. After aging, the product was washed several times for Na^+ ions removal and subsequently dried at 100°C.

Macroporous support was prepared according to the same procedure as mesoporous support except for reverse reagents addition: 3M solution of HNO_3 was added dropwise to the Na_2SiO_3 solution. The pore size distributions for the synthesized supports are shown in Figure S3.

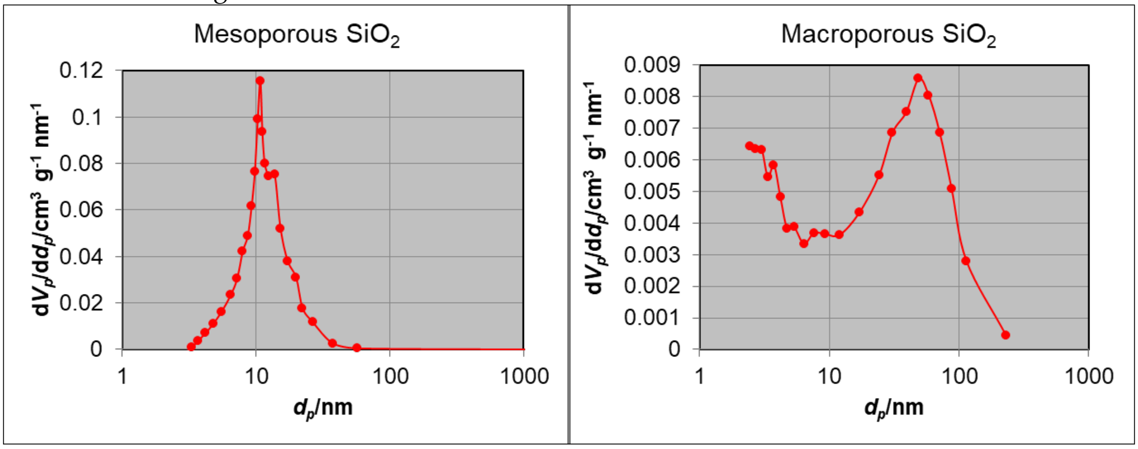

Figure S3. Pore size distribution based on desorption branch of the BJH plot of mesoporous and macroporous supports.

Disclaimer/Publisher's Note: The statements, opinions and data contained in all publications are solely those of the individual author(s) and contributor(s) and not of MDPI and/or the editor(s). MDPI and/or the editor(s) disclaim responsibility for any injury to people or property resulting from any ideas, methods, instructions or products referred to in the content.
